# Rapid Emergence of Free-Riding Behavior in New Pediatric Immunization Programs

**DOI:** 10.1371/journal.pone.0012594

**Published:** 2010-09-15

**Authors:** Chris T. Bauch, Samit Bhattacharyya, Robert F. Ball

**Affiliations:** 1 Department of Mathematics and Statistics, University of Guelph, Guelph, Ontario, Canada; 2 Office of Biostatistics and Epidemiology, Center for Biologics Evaluation and Research, United States Food and Drug Administration, Rockville, Maryland, United States of America; University of Oxford, Viet Nam

## Abstract

**Background:**

Mathematical models have formalized how free-rider effects can threaten the stability of high vaccine coverage levels under established voluntary vaccination programs. However, little research has addressed the question of when free-riding begins to develop when a new vaccine is first introduced in a population.

**Methodology/Principal Findings:**

Here, we combine a game theoretical model of vaccinating behavior with an age-structured compartmental model to analyze rational vaccinating behavior in the first years of a universal immunization program, where a new vaccine is free to all children of a specified age. The model captures how successive birth cohorts face different epidemiological landscapes that have been shaped by the vaccinating decisions of previous birth cohorts, resulting in a strategic interaction between individuals in different birth cohorts. The model predicts a Nash equilibrium coverage level of 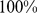 for the first few birth cohorts under the new program. However, free-riding behavior emerges very quickly, with the Nash equilibrium vaccine coverage dropping significantly within 2-5 years after program initiation. Subsequently, a rich set of coupled dynamics between infection prevalence and vaccinating behaviors is possible, ranging from relatively stable (but reduced) coverage in later birth cohorts to wide fluctuations in vaccine coverage from one birth cohort to the next. Individual tolerance for vaccine risk also starts out at relatively high levels before dropping significantly within a few years.

**Conclusions/Significance:**

These results suggest that even relatively new immunization programs can be vulnerable to drops in vaccine coverage caused by vaccine scares and exacerbated by herd immunity effects, necessitating vigilance from the start.

## Introduction

Voluntary vaccination programs–where individuals are free to choose whether to vaccinate–can be victims of their own success. As vaccine-derived herd immunity builds, the probability of being infected declines and eventually goes to zero. However, the perceived risk of vaccination remains constant. Hence, rational individuals may cease to vaccinate once herd immunity is sufficiently strong. This effect has been variously described in terms of a Free-Rider problem, the Prisoner's Dilemma, or the Tragedy of the Commons. It can also be seen as an example of policy resistance, which has been defined as “the tendency for interventions to be defeated by the systems response to the intervention itself” [1].

More broadly, the free-rider problem in vaccination is one possible outcome of the nonlinear interplay between infection prevalence and individual vaccinating behavior. This interplay can be captured by a model consisting of a submodel of disease dynamics and a submodel of individual vaccinating behavior. Such integrated frameworks have been used previously to model phenomena such as free-riding behavior under voluntary vaccination, vaccine scares, and related phenomena [2]–[17]. Recent work in this area has focused on using contact networks to describe disease transmission [12], [14]–[16]; using data to develop models closely tailored to individual vaccines and infections [8], [13]; and introducing new mathematical techniques, greater mathematical rigor, or studying different types of disease natural histories [7], [11], [17], [18].

Some of these approaches use game theory, which predicts what strategies an individual should adopt when their payoff depends on what strategies others adopt. Game theory requires solving for the Nash equilibrium, which in population games is a strategy such that no sufficiently small group of individuals can achieve a higher payoff by adopting a different strategy [19]. In the context of vaccination policy, full vaccination coverage is often predicted not to be a Nash equilibrium, since herd immunity deriving from high vaccine coverage means a small group of individuals could achieve a higher payoff by not vaccinating [5].

Previous models focus on vaccinating behavior in a population without age structure where an immunization program has been in place for a long time, and often ignore age structure with a few exceptions [8]. However, there is a broad class of problems where this does not apply. Very little research in behavior-prevalence modelling has been devoted to the question of how vaccinating behavior evolves in the first years of a free, universal immunization program, where vaccines for a pediatric infectious disease are offered for free at a specified age. While the first few birth cohorts may choose to vaccinate at high rates due to the initial persistence of endemic infection in the population, it is not clear what strategy later birth cohorts will adopt, or when.

This question is very topical given the large number of new generation vaccines that have been introduced in recent years, with many others to be introduced in the future [20]. Being able to understand and predict behavior in the first years of a new immunization program is potentially crucial, not only for being able to pre-empt obstacles to program success, but also for the design of clinical and safety trials and studies associated with the licensing process. For instance, designing the size of phase III clinical trials and phase IV safety studies requires knowing the largest vaccine risk that individuals under a voluntary policy will (or should) tolerate.

A parent's decision to vaccinate a child is often not a simple comparison of the risk/benefit of vaccinating versus not vaccinating, but involves a complex interplay of values with scientific facts. Before trying to understand the effects of this complex interaction, examining how the decisions of a hypothetical perfectly “rational” person who simply weighs the risks and benefits is warranted. By so doing, we can provide a baseline against which to compare “real world” decision making. In addition such a model could provide a framework for decisions about safety study timing and size.

At present decisions about how large to make vaccine safety studies at different phases of vaccine development and use, in the absence of a specific safety concern, are made using rules of thumb derived from expert judgment, experience, and engagement with the public [21]. Often absent from such decisions is an explication of the risk/benefit framework from which such decisions derive. Developing a model-based framework identifies the critical assumptions, that can help focus information gathering, engagement around values, and decision making.

Addressing these issues requires a model that is consistent with the context in which individuals make vaccination choices under a universal immunization program. For example, the framework must be able to capture cohort structure, and in particular how each successive birth cohort faces a different landscape of infection risks depending on the vaccinating decisions that have been made by previous birth cohorts. As a game, this would describe a strategic interaction through time between individuals born in different birth cohorts.

In this paper we develop a game theoretical model of the time evolution of vaccine uptake following the introduction of a newly licensed vaccine at a specified age through a universal immunization program. Vaccination is assumed to be voluntary and free. Disease dynamics in the first years of the program are modeled using an age-structured compartmental transmission model. Our objective is to investigate the evolution of vaccinating behavior when a new vaccine is first introduced. In the following section we describe the behavioral and disease dynamic submodels. In the Results section, we characterize how the Nash equilibrium vaccine coverage varies across different birth cohorts and how the dynamics depend on perceived vaccine risk, disease severity, case importation rate, and vaccine efficacy. We specifically characterize how quickly free-riding behavior starts to develop in the population for a given level of vaccine risk, and how vaccine risk tolerance varies across birth cohorts. We conclude with a Discussion section.

## Methods

### Behavioral submodel

The players of the game are children in birth cohort 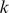, where 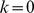 is the first cohort for which vaccine is available under the universal immunization program, 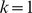 is the second, etc. Players choose at a specified age 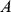 whether or not to vaccinate. (This is a realistic assumption for free school-based immunization programs, where vaccination is scheduled only at a specific age or grade, and it is also a realistic assumption for vaccines that are licensed for use only in a relatively restricted age range, such as for some rotavirus vaccines.) Although it is actually parents who decide whether or not to vaccinate their children, we assume that parents attempt to maximize the payoff to their children. We assume a mixed strategy set, such that individuals in cohort 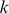 choose to vaccinate with probability 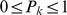.

We denote the vector of strategies played by all cohorts by
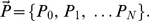
(1)


where 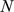 is the time horizon. Likewise, the vector of strategies for all cohorts except 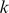 is denoted
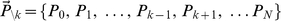
(2)


Each individual in a given cohort receives a baseline payoff 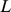 in the absence of disease or vaccine. This can represent, for example, the expected life years or QALYs accrued before risks from the disease or the vaccine are taken into account. We let 
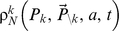
 denote the proportion of non-vaccinated individuals in cohort 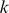 who are infected at age 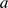 and year 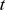 when they adopt strategy 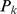 and individuals in other cohorts adopt strategy 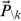. The corresponding notation for the proportion of vaccinated individuals who become infected is 
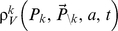
. We also define 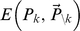 as the payoff to an individual in cohort 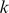 playing strategy 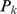, when the other cohorts play strategy 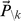. The payoff 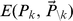 is given by
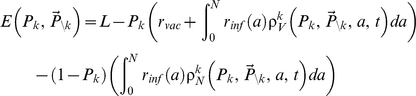
(3)


where 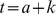, 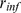 is the penalty for becoming infected, and 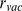 is the penalty for becoming vaccinated.

To define a Nash Equilibrium for this game, consider a population where a fraction 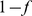 of individuals in a given cohort 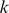 plays strategy 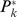 and a fraction 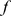 play the alternative strategy 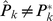. Individuals in other cohorts continue playing 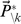. We define the vector of strategies by all cohorts 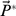 to be a strict Nash equilibrium if
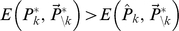
(4)


as 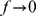 for all 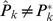 and for all 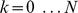. Hence, 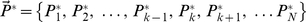 is a strict Nash equilibrium if there is no way a sufficiently small (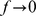) group of individuals in any given cohort 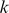 could increase their payoff by switching from strategy 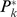 to a different strategy 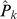, and this must be true for all the cohorts 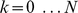.

### Transmission submodel

We computed the quantities 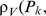 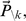 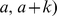 and 
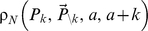
 in Eqn. (3), from the numerical solutions of an age-structured Susceptible-Exposed-Infectious-Recovered-Vaccinated (SEIRV) model, whereby individuals are allocated into one of a number of mutually exclusive categories based on their epidemiological status, age, and strategy. Epidemiological categories were: susceptible vaccinator, susceptible non-vaccinator, exposed vaccinator, exposed non-vaccinator, infectious, removed/recovered, and vaccine immunity. Age classes were: 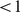 month old, 1 month, 2 months, … , 59 months, 5 years old, 6 years, … , 9 years, 10–14 years old, 15–19 years, … , 75–79 years old.

Susceptible individuals in age class 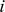 become infected at rate 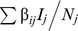, where 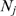 is the total number of individuals of age class 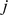 and 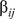 is the rate at which infectious persons of age class 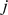 transmit infection to a susceptible person of age class 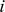. Newly infected individuals remain latently infected for 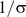 days on average and then enter the infectious class, where they remain for 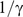 days on average, thereafter acquiring lifelong immunity and entering the removed class. Individuals are born at rate 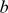. Aging was represented as a discrete time process, by moving individuals from age class 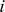 to age class 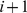 each month, except for a fraction 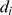 who die due to causes other than complications arising from infection.

Birth cohort 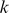 consists of all individuals born in year 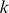. A proportion 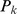 of individuals in birth cohort 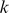 choose to be vaccinated at age 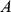. The vaccine efficacy is 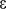. Hence a proportion 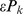 of all individuals in cohort 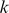 are moved to the vaccine immunity class 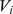 upon reaching age 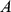. Individuals in the 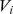 class lose their immunity at rate 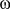, thereby becoming fully susceptible again. We consider the case 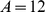 months, and we assume that mortality from infection is small relative to mortality from other causes, as is true for many pediatric infectious diseases in developed countries.

Model parameters were chosen to reflect measles epidemiology. Transmission rates were estimated using a contact surface approach [22], by minimizing the residual sum-of-squares error between simulated force of infection and force of infection estimates from the literature. Other parameters were taken from the epidemiological and modeling literature. The resulting impulsive differential equations appear in online supporting information (Text S1), along with a model diagram and a table of parameter values.

### Determination of Nash equilibria

The Nash equilibrium for the game described by Eqns. (1)–(4) under the SEIRV model dynamics was determined numerically using a simulated annealing algorithm [23]. Initially, a vaccine coverage 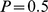 was assigned to each cohort and the SEIRV model was simulated from 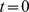 years to 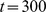 years, with vaccine being introduced at 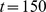 years. Based on the output of this simulation, 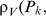 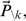 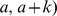 and 
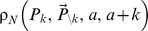
 were computed for each cohort 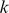. Then, each cohort adjusted its vaccine coverage up or down by a fixed increment according to whether the payoff to vaccinate was higher or lower than the payoff not to vaccinate, and the SEIRV model was simulated again with the newly adjusted vaccine coverages in each cohort 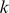.

The process was iterated 1000 times. Every 30 iterations, the vaccine coverage in each cohort was adjusted up or down randomly to prevent convergence to local minima. (In this context, a local minimum represents a solution where the payoff to vaccinate is not as close to the payoff not to vaccinate as another solution elsewhere in parameter space, and therefore globally is not the best Nash equilibrium candidate.) After 1000 iterations, it was confirmed that each cohort 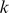 was at a vaccine coverage level 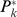 such that the payoff to vaccinate equaled the payoff not to vaccinate. This Nash equilibrium candidate 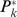 was tested by challenging it with 50 randomly selected alternative strategies played by a small minority of individuals in a single randomly selected birth cohort, and comparing the total payoffs for the small group according to Eqn. (3) versus the payoffs for the remainder in the cohort who continued to play 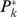.

### Scenarios

We determined Nash equilibria for a range of values of the vaccine penalty 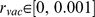, under eight scenarios corresponding to high disease penalties (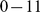 months: 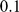, 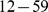 months: 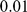, 60+ months: 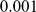) versus low disease penalties (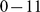 months: 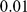, 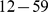 months: 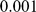, 60+ months: 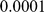), presence of low-level case importation (incidence rate 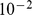 per 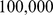 per year) versus absence of case importation, and high vaccine efficacy (95%) versus low vaccine efficacy (70%).

## Results

The first birth cohort for which the vaccine becomes available (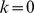) typically faces a situation where the disease is highly endemic. Hence, there is a greater probability of being infected at a young age, where vulnerability to complications of infection is highest (Text S1). Therefore, unless vaccine risk is unacceptably high, we expect the Nash equilibrium strategy to be full vaccination. The second cohort (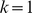) may also opt for full vaccination, although herd immunity provided by the first birth cohort has lessened their risk of infection somewhat. Similar reasoning applies for 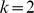. Eventually there is a birth cohort 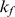 for which a pure vaccinator strategy 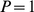 is no longer optimal, due to herd immunity generated by previous vaccinating cohorts. Cohort 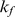 may therefore free-ride on the herd immunity provided by previous vaccinating cohorts. However, this strategy may not be optimal if subsequent cohorts 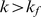 also exempt themselves from vaccination, and/or if any outbreak that results from the non-vaccinating behavior of cohort 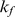 occurs when individuals in cohort 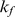 are at an age where infection penalties are still high. Subsequent cohorts 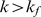 have the disadvantage that, if they do attempt to free-ride by not vaccinating, they will be less protected since the next epidemic is more likely to occur when they are at an age at which disease risk is higher, and there is already less herd immunity for them to rely on due to the non-vaccinating behavior of cohort 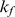. Therefore, subsequent cohorts are more likely to return to a strategy of vaccinating at higher coverage levels, in order to prevent an outbreak that may affect them disproportionately.

**Figure 1:**
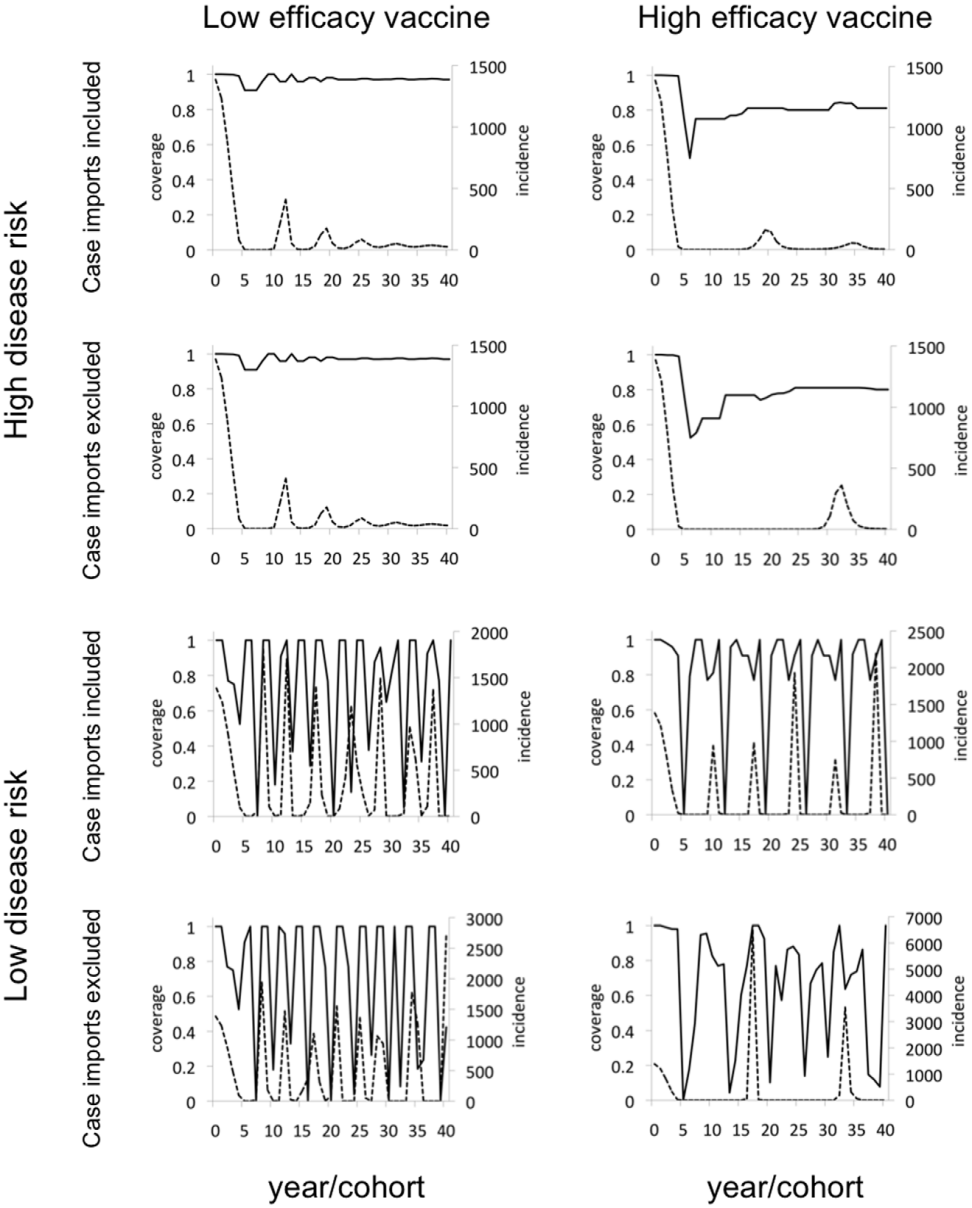
Nash equilibrium vaccine coverage by cohort, and incidence per 100,000 by year, for 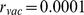 under the 8 scenarios (see main text). Solid line represents coverage, and dashed line represents cases.

This strategic interaction through time between individuals born in different cohorts can result in a rich variety of behavior (Figure 1). However, in every scenario, free-riding behavior develops surprisingly quickly, within the first 2–5 years of the program. In all eight scenarios, Nash equilibrium vaccine coverage is 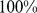 in the first birth cohort. In the scenarios of high disease risk, vaccine coverage also remains close to 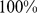 in the first 4–5 birth cohorts; in subsequent cohorts it drops but remains relatively high, with minor adjustments in coverage between cohorts in response to intermittent periods of epidemic activity; disease prevalence is low but non-zero due to free-riding. By comparison, when disease risk is small, vaccine coverage is highly variable from one cohort to the next, resulting in irregular patterns and more frequent outbreaks (Figure 1). In some cases, birth cohorts free-ride by adopting pure non-vaccinator strategies, followed by birth cohorts that adopt pure or intermediate vaccinator strategies. The average vaccine coverage across birth cohorts is also lower for the scenarios of lower disease risk.

**Figure 2:**
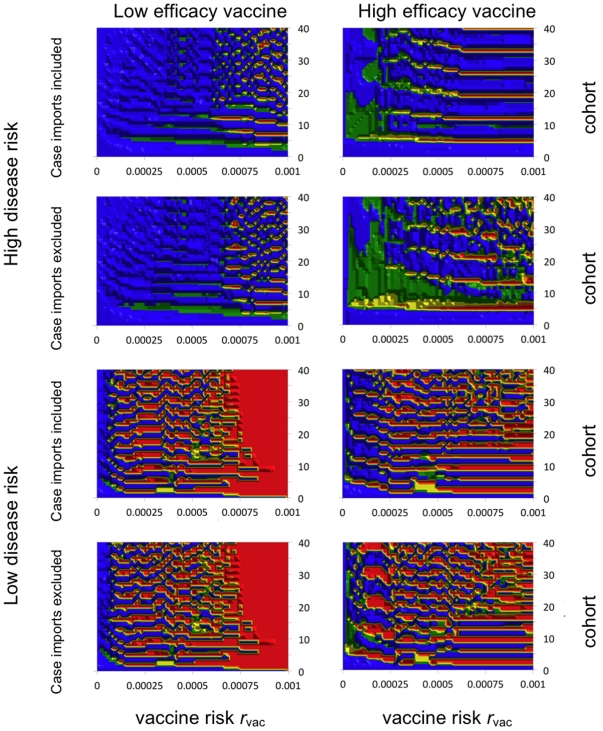
Nash equilibrium coverage levels versus cohort and vaccine risk under the 8 scenarios (see main text). Blue >80% coverage, green 60-80%, yellow 40–60%, orange 20–40%, red 0%–20% coverage.

The model dynamics across the eight scenarios can be visualized by surface plots of the Nash equilibrium coverage 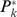 as a function of birth cohort 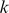 and vaccine risk 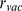 (Figure 2). The destabilizing effect of lower disease risk observed in Figure 1 is also apparent in the surface plots of Figure 2. For very low levels of vaccine risk, vaccine coverage is high and relatively stable across cohorts, whereas increasing levels of vaccine risk quickly destabilize coverage across cohorts and reduce average coverage levels. The depth of non-vaccinating troughs increases as vaccine risk 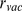 increases, although not necessary their width (number of adjacent non-vaccinating cohorts). The first cohort to reduce its vaccine coverage, which dictates the frequency of non-vaccinating behavior in subsequent cohorts, varies with vaccine risk. The resulting alternation of low and high coverage levels resemble natural patterns such as sand bars or zebra stripes, which are also attributable to nonlinear feedbacks (Figure 2) [24]. The range of vaccine risks analyzed here was chosen to capture the spectrum of possible dynamical behaviors, rather than being based on actual or perceived vaccine risks from real populations.

When disease risk is high, the effect of reduced vaccine efficacy is to increase vaccine coverage (Figure 2). This occurs because a sufficiently imperfect vaccine cannot interrupt disease transmission, even at 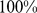 coverage, and thus the persistent risk of infection continues to encourage individuals to vaccinate. However, low vaccine efficacy also makes the vaccine less attractive since it is less likely to protect against infection. This competing effect appears to dominate in the case of low disease risk, where low vaccine efficacy reduces overall coverage without significantly changing the pattern of wide variability in vaccine coverage across birth cohorts.

An aspect of behavior-prevalence dynamics that has previously been neglected is the role of case importation. This is the only source of disease burden once local chains of transmission have been interrupted through herd immunity, therefore, inclusion of case importation should potentially modify predicted vaccinating behavior once herd immunity has been built through immunization programs. In the scenarios of high disease risk in the present model, including case importation stabilizes patterns of vaccine coverage, creating greater consistency in predicted behavior for various values of 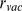. Case importation also increases the frequency of outbreaks moderately.

An important question in the design of phase III vaccine trials and phase IV safety studies is: how small a vaccine risk must be ruled out before the vaccine is considered acceptable for licensing? From the standpoint of an individual's decisions, this question becomes: how small does the vaccine risk have to be for a rational person to decide it is in their interest to vaccinate? The answer to this will depend on the birth cohort, since different birth cohorts face different epidemiological landscapes that have been shaped by the vaccination decisions of older birth cohorts.

**Figure 3:**
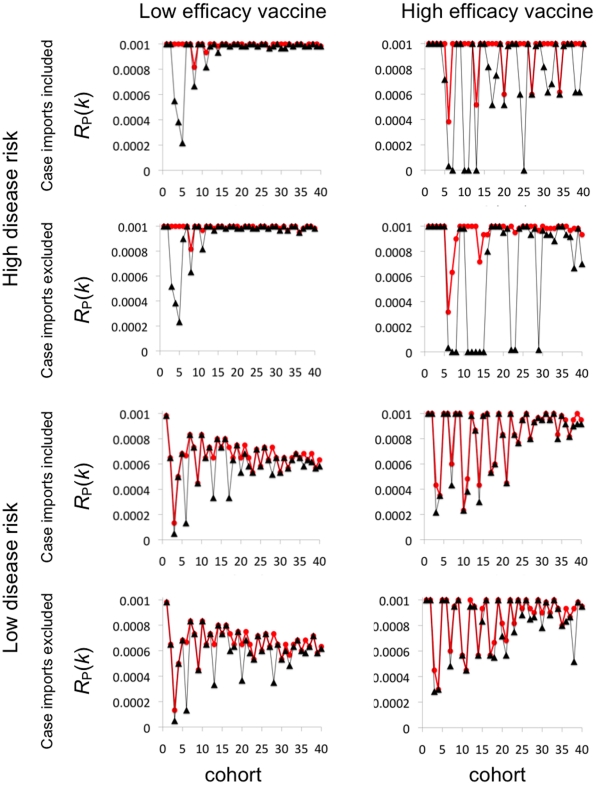
Largest tolerated risk 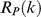 versus cohort k under the 8 scenarios (see main text). Triangles: 95% coverage, circles: 50% coverage.

To address this question, we define 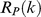 as the smallest vaccine risk for which the Nash equilibrium vaccine coverage in cohort 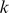 will be at least 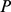, where 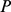 is conceived of as a desirable target coverage by a public health decision maker. The predicted dependence of 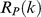 on model parameters (Figure 3) mirrors the dependence of Nash equilibrium coverage 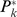 on model parameters (Figures 1 and 2). The first few birth cohorts are generally willing to tolerate a relatively high vaccine risk 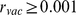. However, subsequent birth cohorts differ considerably in how much vaccine risk they will tolerate, with cohorts that are able to free-ride on herd immunity tolerating lower vaccine risk. In the scenario of high disease risk and low vaccine efficacy with 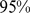 target coverage, the first seven birth cohorts tolerate vaccine risk 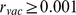. At the other extreme lies the scenario of low disease risk and low vaccine efficacy, where only the first birth cohort is willing to tolerate vaccine risk 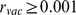.

Lowering disease risk lowers the average tolerance for vaccine risk across birth cohorts, which is not surprising (Figure 3). However, the way that tolerance is distributed across birth cohorts can change in surprising ways as disease risk is lowered. For instance, when vaccine efficacy is high, lower average tolerance takes the form of more cohorts with intermediate tolerance, rather than a few cohorts with zero tolerance. In the scenario of low efficacy vaccine, the decrease in tolerance that occurs as disease risk becomes lower is manifested by decreased vaccine tolerance in all birth cohorts (whereas when disease risk is high, reduced tolerance for vaccine risk is concentrated in early birth cohorts).

Increasing vaccine efficacy also has some surprising and paradoxical effects. In the scenarios of high disease risk, increasing vaccine efficacy decreases tolerance for vaccine risk in most birth cohorts. This occurs because use of a higher efficacy vaccine makes it possible to achieve very low incidence through herd immunity, and the resulting low force of infection reduces the tolerance for vaccine risks. By comparison, in the scenarios of lower disease risk, increasing vaccine efficacy results in widely variable vaccine risk tolerance across cohorts.

There are few qualitative differences in 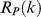 between 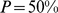 and 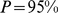 when disease risk is low. However, when disease risk is high, the largest tolerable vaccine risk is much smaller (sometimes close to zero) for 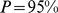 than for 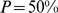. An exception occurs in the case of low vaccine efficacy and high disease risk, where the tolerated vaccine risk remains high only in the first two cohorts when 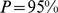, but the tolerated vaccine risk remains high in the first seven cohorts when 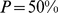 (Figure 3).

The social optimum was defined as the vaccine coverage 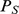 such that the total burden in all cohorts from either vaccine or infection is minimized, and was was assumed to be the same in all cohorts. When vaccine risk is zero, 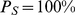 (Text S1). As vaccine risk increases, the 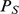 drops below 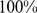, since additional vaccination beyond the herd immunity threshold does not prevent significant additional infection but does impose some burden due to vaccine risks. 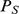 declines with increasing vaccine risk, but not always monotonically. In some cases, it is constant and then declines abruptly with increasing vaccine risk. This occurs because of assumptions about decreasing disease risk with increasing age.

## Discussion

Here we developed a game theoretical model of vaccinating behavior that is closely tailored to the way that many vaccines are implemented in real populations, through free, universal programs that offer vaccine to all children at a recommended age. Using a compartmental measles model to capture the response of disease dynamics to vaccination choices, we modeled the strategic interaction between individuals in the same birth cohort as well as individuals in different birth cohorts. We studied the resulting transient solutions of behavior-prevalence dynamics in the first years after a new vaccine has been introduced into the population. This framework built in the realistic cohort structure that is an integral part of many publicly-financed, school-based immunization programs, and thus the framework captures how successive birth cohorts face different epidemiological conditions, especially in the first years of an immunization program. This situation is highly relevant for many new generation vaccines that have been recently introduced (such as new generation rotavirus vaccines) or that will eventually be introduced. The design of this approach was based on a desire to address issues that arise in the design of vaccine licensing protocols, where one must decide how large a vaccine risk is acceptable, either from the viewpoint of a decision-maker or from the viewpoint of a rational individual under a voluntary vaccination policy.

Free-riding behavior under voluntary vaccination policies is widely expected for long-established immunization programs where significant levels of herd immunity derived from immunization have been built up. This potential instability in long-established immunization programs has been captured by many previous models [2]–[11], [13], [17], [25], [26]. By comparison, it is not clear how quickly free-riding should emerge when a vaccine has been introduced for the first time. Here we found that free-riding can develop surprisingly quickly, within the first 2–5 years of a new voluntary immunization program (Figures 1 and 2).

More generally, we found that vaccine efficacy, disease risk, and vaccine risk can interact to result in a rich and often surprising variety of possible vaccine coverage and outbreak patterns at epidemiologically plausible parameters values. These patterns range from relatively high, stable vaccine coverage across all cohorts with occasional outbreaks (for relatively low disease risk) to extremely variable vaccine coverage from one cohort to the next with regular outbreaks (for relatively high disease risk).

We also found that the tolerance for vaccine risk depends on the birth cohort. Earlier birth cohorts are subject to higher levels of infection prevalence and hence are more willing to tolerate higher risks and vaccinate at higher levels of coverage. However, the tolerance for risk drops dramatically at some point within the first five birth cohorts. This drop occurs sooner for lower disease risk and/or lower vaccine efficacy. For the case of high efficacy vaccine and high disease risk, this drop does not occur until the fifth birth cohort vaccinated, under the 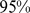 coverage target. (However, subsequent cohorts may increase coverage, often up to 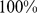 again, in order to stave off epidemics that would generate significant disease burden for them.)

These findings indicate that sample size calculations for phase III vaccine trials and database size determination for phase IV safety studies should consider that risk tolerance can change during a vaccine rollout campaign. The simulation results suggest that for vaccine-disease systems where disease risk and vaccine efficacy are sufficiently high, there may be a window during the early years of an immunization program where individuals are willing to tolerate greater uncertainty in vaccine risk. All other things being equal, one might therefore argue that shifting more safety data collection to the time after the vaccine is introduced into use is a rational policy. As a result, large phase III trials may not be needed, although a large-scale safety study may be needed post-licensure because tolerated risk may drop significantly after a few years. The present analysis demonstrates how modeling this interaction between vaccine risk tolerance, vaccine acceptance, and infection rates in the presence of vaccination can provide a baseline to quantitatively evaluate current licensing and safety monitoring strategies, as well as a method of computing required sample sizes for clinical trials and safety study database size. The practicality of such an approach would need to be assessed. For instance, enrolled patients are sometimes lost to follow-up and/or can be very poor at self-diagnosing adverse events. Such factors can complicate measurement of vaccine risk and thus required sample size.

Our analysis assumed individuals have perfect rationality. Normally, this may be considered to be a limitation of classical game theoretical models. However, for the question of informing vaccine licensing protocol, rationality is actually a useful assumption. For the purposes of vaccine licensing, regulators wish to determine the required size of phase III vaccine safety trials. An important criterion in determining the required size of phase III trials is the need to rule out vaccine adverse events up to a certain acceptable probabilityin other words, there is a need to determine largest acceptable vaccine risk. The largest acceptable vaccine risk could be determined by several criteria. However, one criterion is the level of vaccine risk that a self-interested but rational person would be willing to tolerate, given information on disease risks and epidemiology. By comparison, how actual irrational individuals will react to a new vaccine is arguably not a good basis for deciding whether or not a vaccine should be licensed, because licensing decisions should be based on scientific data.

On the other hand, for other types of questions, such as predicting actual behaviour upon introduction of a new vaccine, the assumption of perfect rationality could be a significant limitation. Game theoretical models that assume perfectly rationality tend to predict more irregular behaviour (such as oscillations) for certain parameter regimes than are observed in reality. Hence, if more realistic human behavior is introduced into this framework, there may be greater stability of vaccine coverage across cohorts. This model was also developed for measles-like infection and vaccine. Infections other than measles may exhibit more regular patterns or may exhibit less extreme oscillations, since measles transmission is significantly nonlinear. The wide range of dynamical behavior seen in this model emphasizes the need to understand the impact of our model assumptions on the predicted dynamics. Future work could investigate how relaxing these assumptions influences predictions under this framework. Other future applications of this framework might involve case studies for specific disease/vaccine systems. One such example might be to explore whether the general acceptance of MMR vaccine despite known adverse effects such as idiopathic thrombocytopenic purpura (ITP) [27], is consistent with the predictions of this model.

As noted above, this model suggested that free-riding behavior can emerge relatively soon after a new immunization program for measles-like infection has been introduced. This is true even before vaccine-derived herd immunity has been built up in a large proportion of the overall population. This prediction may hold for a variety of disease systems. Mixing between age classes–both in terms of social contacts and in terms of sexual contacts–tends to be highly assortative [28], [29]. Hence, the herd immunity effects of a newly introduced vaccination program are likely to be stronger in age classes that are close to the age at which vaccination is scheduled. As a result, free-riding behaviour may threaten not just well-established immunization programs but also new ones. This also means that populations can be vulnerable to vaccine scares within a few years after a new vaccine for a pediatric infectious disease has been introduced. Hence, further study of the interaction between vaccinating behavior and disease dynamics under new immunization programs is needed.

## Supporting Information

Text S1 Additional information on model equations, parameterization, and supplemental results.
(2.62 MB PDF)
