# Supplementary material for: Rapid Emergence of Free-Riding Behavior in New Pediatric Immunization Programs
Source: PLoS One. 2010 Sep 15;5(9):e12594. doi: 10.1371/journal.pone.0012594 (PMC2939872; doi:10.1371/journal.pone.0012594)
Supplement: Text S1 — Additional information on model equations, parameterization, and supplemental results. (2.62 MB PDF) [file pone.0012594.s001.pdf]

Supporting Information for

"Rapid emergence of free-riding behavior in new pediatric immunization programs"

C.T. Bauch, S. Bhattacharyya, R. Ball

August 12, 2010

Table of Contents

Appendix A1: Model description …………………………………………………………. 1
1.1. Vaccination program…………………………………………………………. 1
1.2. Game assumptions…………………………………………………………… 2
1.3. Transmission model………………………………………………………….. 3
1.4. Computing payoffs from transmission model………………………………… 7
1.5. Determination of Nash equilibrium…………………………………………... 8
Appendix A2: Supplementary results……………………………………………………… 9
Figure A3: Social optimum…………………………………………..………… 9
Figure A4: Minimum coverage in any cohort …………………………………… 10
References……………………………………………………………………………….. 11

Appendix A1. Model Description

1.1 Vaccination Program

We consider universal vaccination implemented in children 12 months of age, with no catch-up vaccination. Vaccination is voluntary.

1.2 Game Assumptions

We assume that players are perfectly rational: players have perfect information and try to maximize their payoff, i.e., minimize lifetime health risks from either vaccine or disease. The assumption of rationality provides a benchmark against which to compare actual behavior. Players choose at a specified age A whether or not to vaccinate, and they cannot vaccinate before or after that age. Hence, each birth cohort must consider at age A whether or not to vaccinate.

Players: The players are individuals in birth cohort k, where k=0 is the first cohort to be vaccinated under the universal program, k=1 is the second cohort, etc. (Note that for pediatric

vaccines, the players will technically be children but parents make decisions). All birth cohorts have the same decision-making processes. We assume that the age at which individuals are vaccinated is A. We assume a population game where the payoff to an individual depends on the average behavior in the population.

<u>Strategy Set</u>: Individuals in cohort k adopt strategy P, where $0<P<1$ is the probability to vaccinate (we assume that individuals in the same cohort behave identically and hence all vaccinate with the same probability). Hence, the strategy set is

$$\{P : 0 \le P \le 1,\ P \text{ real}\} \tag{1}$$

For each cohort k we seek its Nash equilibrium strategy P_k^*. We expect $P_k^*=1$ to be the Nash equilibrium for the earliest cohorts (k small), and subsequently $P_k^*<1$ when herd immunity is stronger. We denote the strategy of cohort k by P_k, and the vector of strategies for all other cohorts is given by

$$\vec{P}_{\backslash k} = (P_0, P_1, \ldots, P_{k-1}, P_{k+1}, \ldots, P_{80}) \tag{2}$$

Likewise we denote the vector of all strategies by

$$\vec{P} = (P_0, P_1, \ldots, P_{80}) \tag{3}$$

<u>Payoffs</u>: Each individual in a given cohort receives a baseline payoff L in the absence of disease or vaccine. This can represent, for example, the expected life years or QALYs accrued before risks from the disease or the vaccine are taken into account. Vaccination at age A incurs a penalty r_{vac} due to adverse events from vaccination. Likewise, contracting infection incurs a penalty $r_{\text{inf,N}}(a)$ in non-vaccinated individuals and $r_{\text{inf,V}}(a)$ in individuals unsuccessfully vaccinated. We let $\rho_N(P_k, \vec{P}_{\backslash k}, a, t)$ denote the proportion of non-vaccinated individuals in cohort k who are infected at age a and year t when their strategy is P_k and individuals in other cohorts play strategy $\vec{P}_{\backslash k}$. The corresponding notation for vaccinated individuals who become infected is $\rho_V(P_k, \vec{P}_{\backslash k}, a, t)$. We also define $E(P_k, \vec{P}_{\backslash k})$ as the payoff to an individual in cohort k playing strategy P_k, when the other cohorts are playing strategy $\vec{P}_{\backslash k}$. This is given by the equation (where $t=a+k$)

$$E(P_k, \vec{P}_{\backslash k}) = L - P_k\left(r_{vac} + \int_0^{80} r_{\text{inf},V}(a)\rho_V(P_k, \vec{P}_{\backslash k}, a, a+k)da\right) - (1-P_k)\int_0^{80} r_{\text{inf},N}(a)\rho_N(P_k, \vec{P}_{\backslash k}, a, a+k)da \tag{4}$$

We assume that vaccine efficacy does not depend on whether the vaccinee experiences adverse events.

Nash Equilibrium: To find the Nash equilibrium for this game, consider a population where a fraction 1-f of individuals in cohort k plays strategy P_k^* and a fraction f play the alternative strategy $\hat{P}_k \neq P_k^*$. We define $\vec{P}^*$ as a strict Nash equilibrium if

$$E(P_k^*, \vec{P}_{\backslash k}) > E(\hat{P}_k, \vec{P}_{\backslash k}) \text{ as } f \to 0 \text{ for all } \hat{P}_k \neq P_k^* \tag{5}$$

holds for all choices of k. In other words, $\vec{P}^*$ is a strict Nash equilibrium if there is no way a sufficiently small ($f \to 0$) group of individuals in any cohort could increase their payoff by switching to a different strategy ($E(P_k^*, \vec{P}_{\backslash k}) > E(\hat{P}_k, \vec{P}_{\backslash k})$ for all $\hat{P}_k \neq P_k^*$), and this must be true for all the cohorts.

To estimate payoff functions, we need to estimate future risk to vaccinated and unvaccinated individuals. Perfect information in the context of vaccinating for an infectious disease is taken to mean that individuals have perfect information on future prevalence of infection under both Nash and potential non-Nash equilibrium scenarios. This means they make plausible assumptions about future infection risks based on best epidemiological knowledge, i.e., SEIR model projections.

1.3. Transmission model

To compute payoffs under various possible vaccination strategies, we use a modified age-structured SEIRV model, whereby individuals are allocated into one of a number of mutually exclusive categories based on their epidemiological status and age. This allows us to determine future risks for individuals adopting various strategies.

Epidemiological categories were: susceptible (vaccinator or non-vaccinator), exposed (vaccinator or non-vaccinator), infectious, recovered, and vaccinated. Age classes were: <1 month old, 1 month old, 2 months old, …, 59 months old, 5 years old, 6 years old, …, 9 years old, 10-14 years old, 15-19 years old, …, 75-79 years old. Demographic processes (births, aging, death due to causes other than measles) were described using a discrete-time formulation, i.e., at a specific time every month, individuals died due to causes other than measles, aged, or were born and compartment sizes were changed to reflect this. A discrete-time (impulsive DE) formulation was used because artifacts can be introduced by assuming individuals leave an age class at a constant rate. (Between monthly demographic updating of the compartments, ordinary differential equations describe the epidemiological dynamics, as described below.)

A number $b(t)$ of individuals is born each year t. Individuals are born either as susceptible vaccinators or susceptible nonvaccinators, according to their parents' strategy. Each month, individuals of age class i move to age class i+1 (i.e., they age by one month), except for a proportion $d_i(t)$ who die due to causes other than measles.

A discrete-time formulation was also used to describe vaccination. A proportion ε of vaccinees enter the V_i class (ε is the vaccine effectiveness) while 1-ε remain susceptible. The discrete-time equations governing death and aging each year for variable X_a are:

$$X_a(t+1) = (1 - d_{a-1})X_{a-1}(t) \tag{6}$$

where t=time, in months and d_i is the proportion of individuals dying each month due to causes other than measles. Hence, the mappings described by Equation (8) are applied to the model variables (compartment sizes) each year. Births are governed by the equations

$$\begin{aligned} S_0(t+1) &= b_t(1 - P^*) \\ S_0^{vac}(t+1) &= b_t P^* \end{aligned} \tag{7}$$

where b_t is the total number of reported/projected births during month t. Again, these mappings are applied to the model variables (compartment sizes) each month. As mentioned above, epidemiological transitions are described using differential equations. Susceptible individuals in age class i become infected at rate $\lambda_i = \sum_{j=1}^{80} \beta_{ij} I_j / N_j$, where N_j is the total number of individuals of age class j and β_{ij} is the rate at which an infectious person of age class j transmits to a susceptible person of age class i. Newly infected individuals remain latently infected for 1/σ days on average and then enter the infectious class, where they remain for 1/γ days on average, thereafter acquiring lifelong immunity and entering the R_i class. Individuals in the vaccinated class lose their immunity at rate ω, thereby becoming fully susceptible again. Figure A1 presents a schematic diagram of the disease natural history of the model. The epidemiological processes were represented by the equations

$$\begin{aligned}
\frac{dS_i}{dt} &= -S_i \sum_{j=1}^{80} \beta_{ij} \frac{I_j}{N_j} \\
\frac{dS_i^{vac}}{dt} &= \omega V_i \;\; - S_i^{vac} \sum_{j=1}^{80} \beta_{ij} \frac{I_j}{N_j} \\
\frac{dE_i^{vac}}{dt} &= S_i^{vac} \sum_{j=1}^{80} \beta_{ij} \frac{I_j}{N_j} - \sigma E_i^{vac} \\
\frac{dE_i}{dt} &= S_i \sum_{j=1}^{80} \beta_{ij} \frac{I_j}{N_j} - \sigma E_i \\
\frac{dI}{dt} &= \sigma E_i - \gamma I_i + \sigma E_i^{vac} - \gamma I_i^{vac} \\
\frac{dR_i}{dt} &= \gamma I_i + \gamma I_i^{vac} \\
\frac{dV_i}{dt} &= \text{-}\omega V_i
\end{aligned} \tag{8}$$

where the subscript *i* denotes age, and variable definitions are given in Table A1, and parameters are defined in Table A2. We note that $N_j = S_j + S_j^{vac} + E_j + E_j^{vac} + I_j + R_j + V_j$ Also, note that birth, death and vaccine processes are modelled using the discrete time process described previously and hence do not appear in these equations. We keep separate track of exposed and infected vaccinated and non-vaccinated individuals so that the probability of infection, and hence payoff, can be computed for vaccinators versus nonvaccinators. The values of β_{ij} were determined by computing a cumulative percent infected as a function of age from the force of infection by age in Table A2 and minimizing the residual sum of squares error between the resulting empirical profile of cumulative percent infected by age and the modeled profile of cumulative percent infected by age. The values of β_{ij} were constrained by using a contact surface approach (Farrington & Whitaker 2005) where the contact surface was of the form

$$\beta(x,y) = \frac{\kappa u^{k-1} e^{-u/\theta} e^{-d|v|}}{\theta^k (k-1)!}$$

where $u = (x+y)/\sqrt{2}$ and $u = (x-y)/\sqrt{2}$ and where the parameters κ, θ, d and k were fitted. The resulting matrix of β_{ij} values are visualized in Figure A2, where 5-year intervals are used instead of the modelled age classes, to aid visualization.

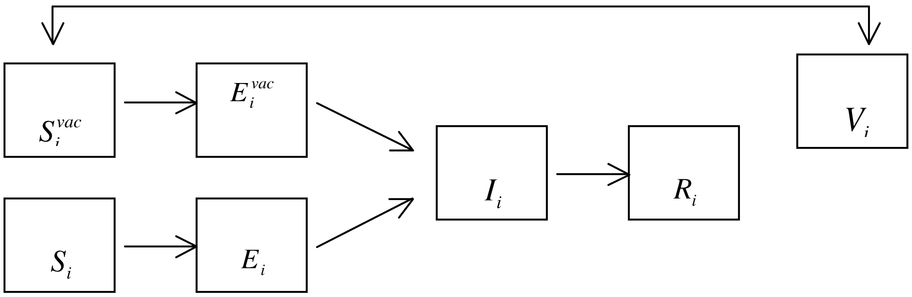

Figure A1: Schematic of natural history component of compartmental model. Arrows represent epidemiologic processes.

Table A1: SEIRV model variables

Variable	Definition
S_i	Number of susceptible non-vaccinators, age class *i*
S_i^{vac}	Number of susceptible vaccinators, age class *i*
E_i	Number of latently infected non-vaccinators, age class *i*
E_i^{vac}	Number of latently infected vaccinators, age class *i*
I	Number of infectious persons, age class *i*
R	Number of recovered persons, age class *i*
V_i	Number of individuals with vaccine-derived immunity, age class *i*

Table A2: SEIRV model parameters

Parameter	Value	Source
Force of infection (used to determine b_{ij})	0-11 months: 0.08<br>12-23 months: 0.10<br>24-35 months: 0.12<br>36-47 months: 0.14<br>48-59 months: 0.16<br>5-9 years: 0.35<br>10-14 years: 0.20<br>15-19 years: 0.14<br>20+ years: 0.07	(Anderson and May 1991)
Duration of latent period ($1/s$)	9 days	(AAP 2006; Collier and Oxford 2006)
Duration of infectiousness ($1/g$)	5 days	(Earn, Rohani et al. 2000; AAP 2006; Collier and Oxford 2006)
Vaccine risk (r_{vac})	Varied from 0 to 0.001	
Vaccine schedule	1 dose at 12 months of age	Assumption
Rate of waning vaccine-derived immunity (w)	1.5% per annum	(Mossong, Nokes et al. 1999; Kremer, Schneider et al. 2006)
Birth rate (b)	100,000 births per annum	Assumption
Death rate per 100,000 per year due to all causes (d_i)	0-11 months: 800<br>12-23 months: 750<br>24-59 months: 38<br>5-14 years: 21<br>15-24 years: 90<br>25-34 years: 126<br>35-44 year: 221<br>45-54 years: 445<br>55-64 years: 1,094<br>65-74 years: 2,538<br>75+ years: 5,803	US Census Bureau
Vaccine efficacy (e)	Varies according to scenario (see main text)	
Disease risk (r_{inf})	Varies according to scenario (see main text)	
Case import rate	Varies according to scenario (see main text)	

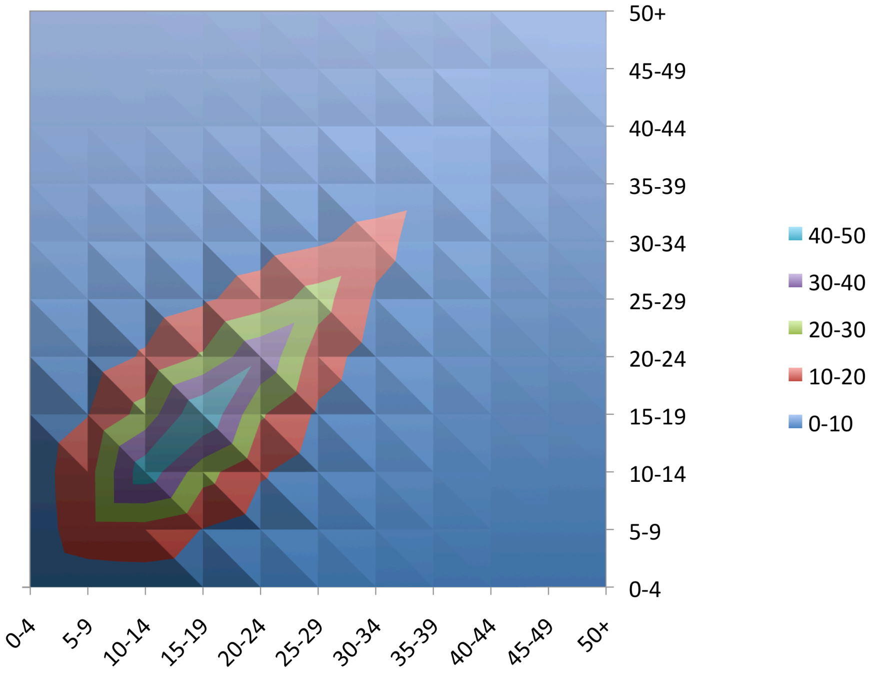

Figure A2: Fitted contact surface using 5-year age classes.

1.4. Computing payoffs from transmission model

Each individual in a cohort receives a baseline payoff L. The compartmental model is used to determine how often measles-relevant outcomes occur for individuals in a given cohort, and for each outcome that occurs, the vaccination and infection penalties are deducted from L. The two events (infection and vaccination) that cause penalties are listed in Table A4 below, along with the model variables that correspond to the events. A limitation of the current model structure assumes equal weighting of infection and adverse events and does not offer the opportunity to code severity and incidence separately. However, modifying the model framework with respect to this is possible.

The payoff $E(P_k, \vec{P}_{\setminus k})$ to an individual in cohort k playing P_k is therefore computed in terms of model variables as

$$E(P_k, \vec{P}_{\setminus k}) = L - P_k\left(r_{vac} + \sum_{a=1}^{80} r_{\inf,V}(a)\frac{\hat{I}_{k,a}^{vac}}{N_{k,a}^{vac}}\right) - (1-P_k)\sum_{a=1}^{80} r_{\inf,N}(a)\frac{\hat{I}_{k,a}}{N_{k,a}} \qquad (9)$$

where age a is between 1 and 80, and N_k is the number of unvaccinated individuals in cohort k. (note that the model variables $\hat{I}_{k,a}$, $\hat{I}_{k,a}^{vac}$, are implicitly a function of the strategies).

Finally, the incidence variables for computing infection penalties are obtained from

$$\begin{aligned}
\hat{I}_{k,a} &= \int_{t=k+a}^{t=k+a+1} \sigma E_a(t)\, dt \\
\hat{I}_{k,a}^{vac} &= \int_{t=k+a}^{t=k+a+1} \sigma E_a^{vac}(t)\, dt \\
\hat{N}_{k,a} &= N_a(a+k)(1-P) \\
\hat{N}_{k,a}^{vac} &= N_a(a+k)P
\end{aligned} \qquad (10)$$

As a future extension, we may determine anticipated future risk according to some phenomenological function that is based on what we know about declining age-stratified infection risk over time and that describes how rational individuals use best available epidemiological knowledge at the time to decide (Fine & Clarkson 1986).

1.5. Determination of Nash equilibria

To identify candidates for the Nash equilibrium, the SEIR model was run repeatedly using a simulated annealing algorithm. An initial vaccine coverage of *P*=0.5 was assigned to each cohort. Under this vaccine coverage, the payoff to vaccinate was compared to the payoff not to vaccinate for each cohort, and the strategies were adjusted accordingly. For instance, if the payoff to vaccinate exceeded the payoff not to vaccinate in a given cohort, then the vaccine coverage was adjusted upward in that cohort. Then, the SEIR model was simulated again and the process was repeated. For cohort *m*, the vaccine coverage P_m^* for which the payoff to vaccinate equals the payoff not to vaccinate was taken as the candidate for the Nash equilibrium. To ensure that the algorithm converged to the Nash candidate, the vaccine coverage in each cohort was periodically disturbed to prevent convergence to a local minimum (simulated annealing). This Nash equilibrium candidate P_m^* was tested by challenging it with randomly selected alternative strategies being played by a small group in the population and comparing the total payoffs for the small group versus the remainder who continued to play the Nash equilibrium strategy.

Table A4: Two events that incur penalties.

Event	Penalty	Relevant Model-derived Quantities	Definition of Model Variables
Infection	$r_{\text{inf,V}}(a)$<br>$r_{\text{inf,N}}(a)$	$\hat{I}_{k,a}^{vac}$,<br>$\hat{I}_{k,a}$	Number of unvaccinated individuals in cohort *k* who are infected at age *a*; number of unsuccessfully vaccinated individuals in cohort *k* who are infected at age *a*
Vaccination	r_{vac}	n/a	n/a

Appendix A2. Supplementary Results

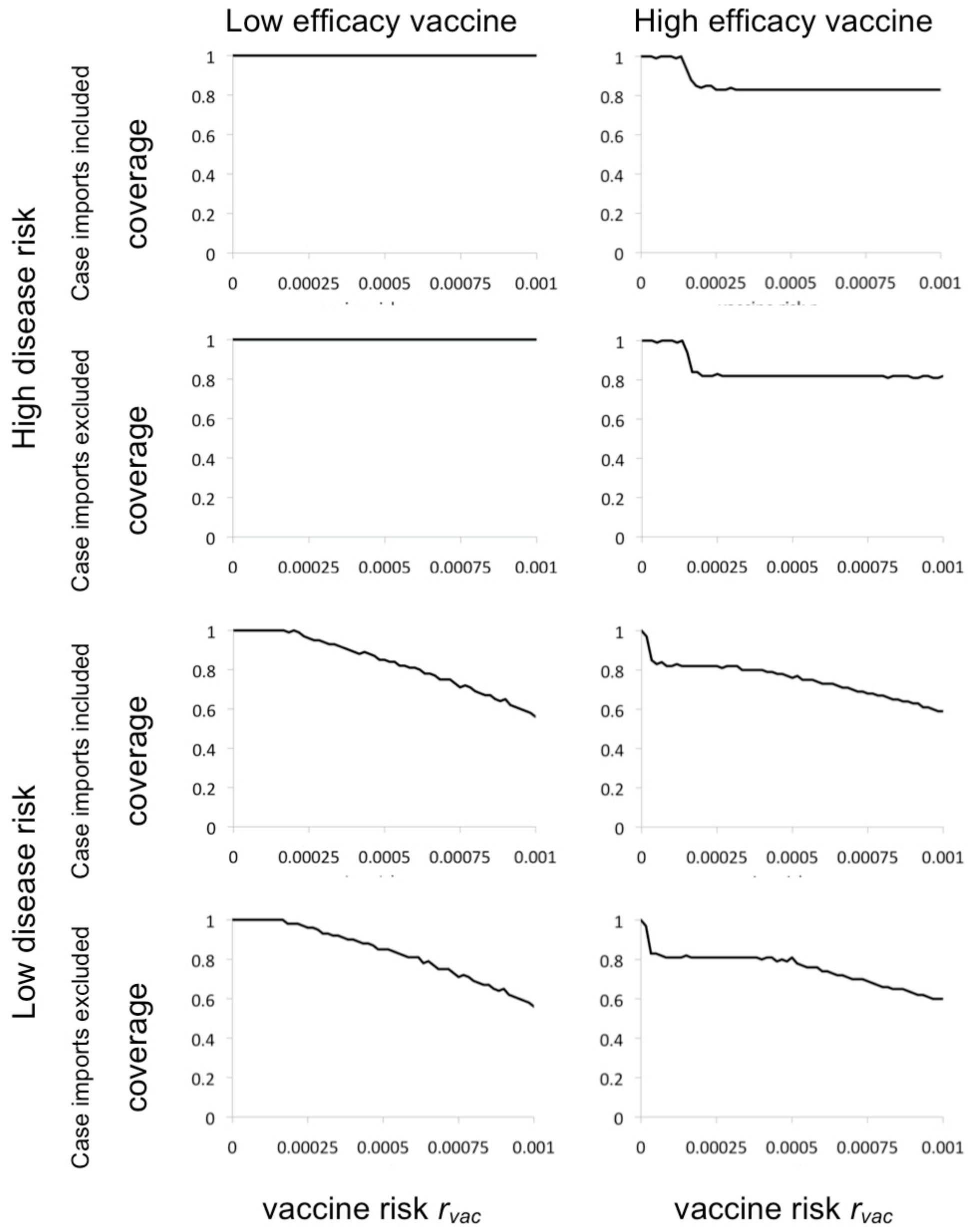

Figure A3: Socially optimal vaccine coverage versus vaccine risk, for 8 scenarios.

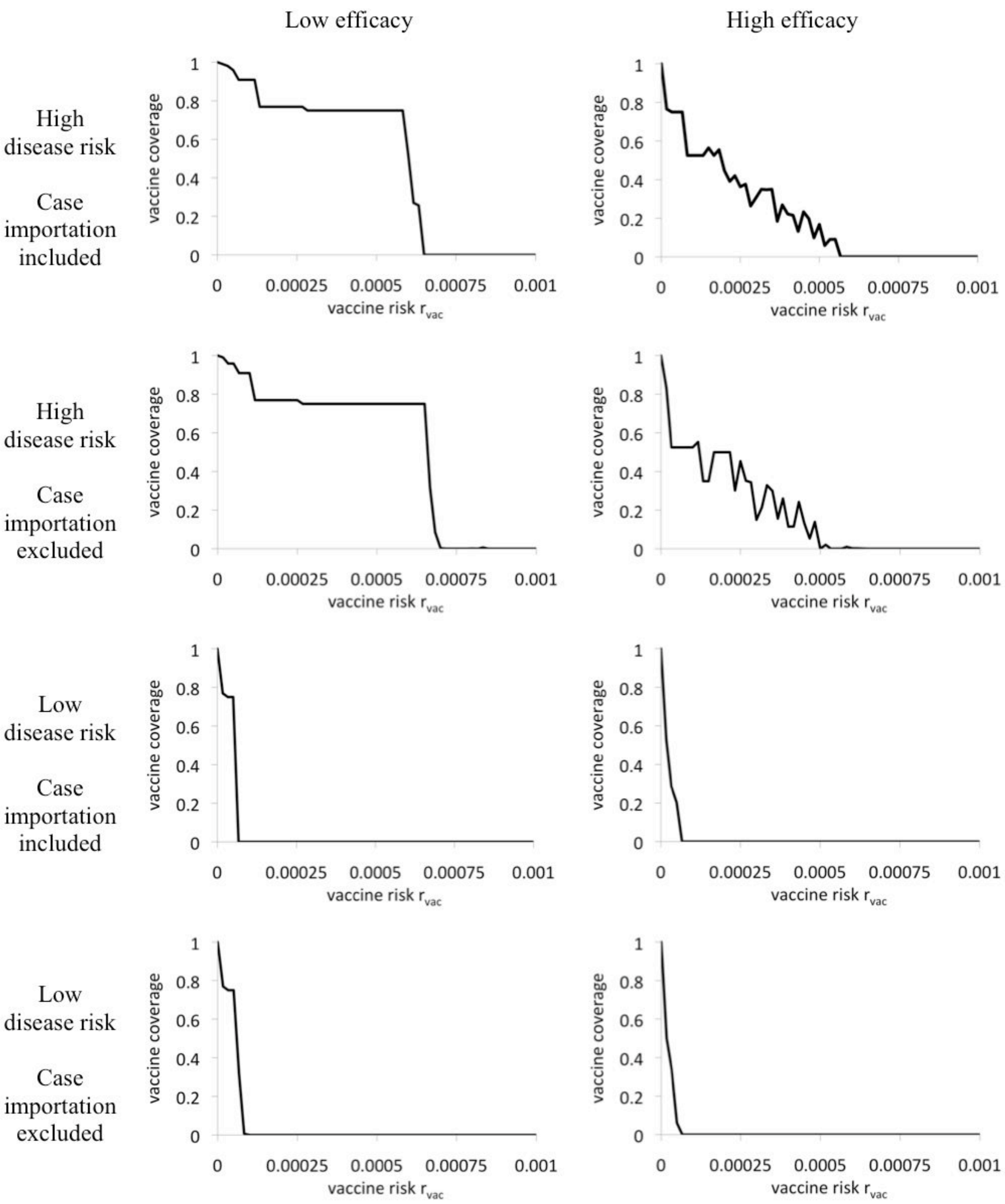

<u>Figure A4</u>: Minimum coverage in any cohort at Nash equilibrium versus vaccine risk, for 8 scenarios.

References

AAP (2006). Measles. Red Book: 2006 Report of the Committee on Infectious Diseases. L. Pickering. Elk Grove Village, IL, American Academy of Pediatrics**:** 441-452.

Anderson, R. and R. May (1991). Infectious diseases of humans. Oxford, Oxford University Press.

Collier, L. and J. Oxford (2006). Human Virology, Oxford University Press.

Earn, D., P. Rohani, et al. (2000). "A simple model for complex dynamical transitions in epidemics." Science **287**: 667-670.

Farrington, C.P., and Whitaker, H.J. (2005). "Contact surface models for infectious diseases: estimation from serologic survey data".

Fine, P. and J. Clarkson (1986). "Individual versus public priorities in the determination of optimal vaccination policies." Am J Epidemiol **124**(6): 1012-1020.

Kremer, J., F. Schneider, et al. (2006). "Waning antibodies in measles and rubella vaccinees--a longitudinal study." Vaccine **24**: 2594-2601.

Mossong, J., D. Nokes, et al. (1999). "Modeling the impact of sublinical measles transmission in vaccinated populations with waning immunity." American Journal of Epidemiology **150**(11): 1238-1249.
